# Memantine augmentation of sertraline in the treatment of symptoms and executive function among patients with obsessive-compulsive disorder: A double-blind placebo-controlled, randomized clinical trial

**DOI:** 10.1192/j.eurpsy.2023.1967

**Published:** 2023-07-19

**Authors:** S. Askari

**Affiliations:** psychiatry, Tehran psychiatry Institute, Tehran, Iran, Islamic Republic Of

## Abstract

**Introduction:**

Medications currently recommended for the treatment of Obsessive-Compulsive Disorder (OCD) usually decrease the severity of the symptoms by 20–30%; however, 40–60% of OCD patients do not achieve a satisfactory response. Our main objective was to investigate the effectiveness of memantine, a non-competitive N-Methyl-D-aspartate (NMDA) receptor antagonist, as an adjunct therapy to sertraline, a selective serotonin reuptake inhibitor (SSRI), to improve severity of symptoms and executive function among patients with obsessive-compulsive disorder.

**Objectives:**

Our main objective was to investigate the effectiveness of memantine, a non-competitive N-Methyl-D-aspartate (NMDA) receptor antagonist, as an adjunct therapy to sertraline, a selective serotonin reuptake inhibitor (SSRI), to improve severity of symptoms and executive function among patients with obsessive-compulsive disorder.

**Methods:**

Seventy patients with OCD according to the Diagnostic and Statistical Manual of Mental Disorders (DSM–5) criteria, and a Yale-Brown obsessive compulsive scale (Y-BOCS) score of more than 21 were recruited to the study. They received sertraline (100 mg daily initially followed by 200 mg daily after week 4) and either memantine (10 mg twice daily) or placebo in a placebo controlled, double-blinded, parallel-group, clinical trial of 12 weeks. The primary outcome was OCD symptoms measured by the Y-BOCS. Moreover, executive function of participants was measured by the Wisconsin Card Sorting Test (WCST).

**Results:**

The total score, and obsession and compulsion subscales of Y-BOCS significantly dropped in both groups with no significant difference between the two groups. However, memantine group showed a greater response in the number of completed categories subscale of the WCST (p value<0.001). We did not observe any major adverse effects in any of the groups.

**Image:**

**Figure:**
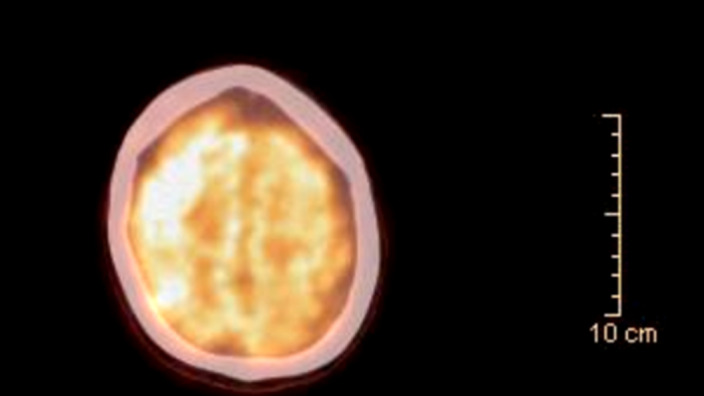

**Image 2:**

**Figure:**
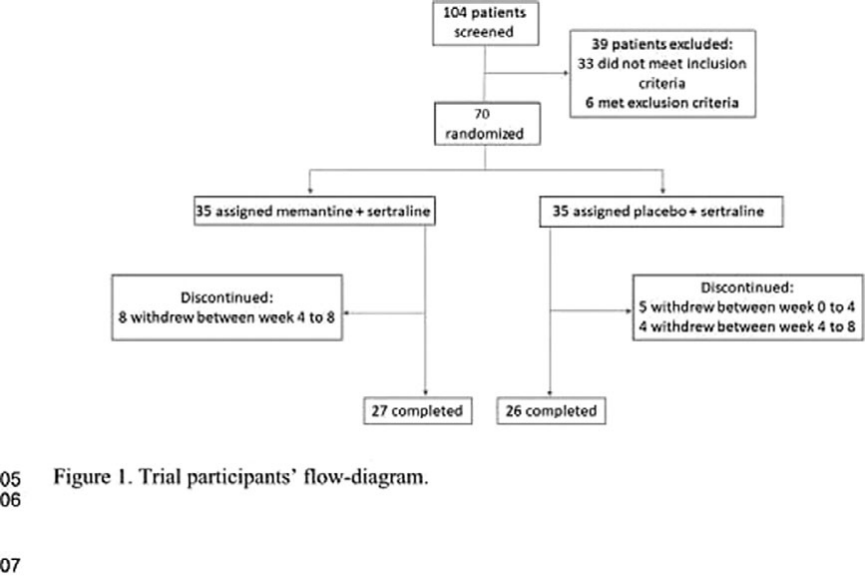

**Image 3:**

**Figure:**
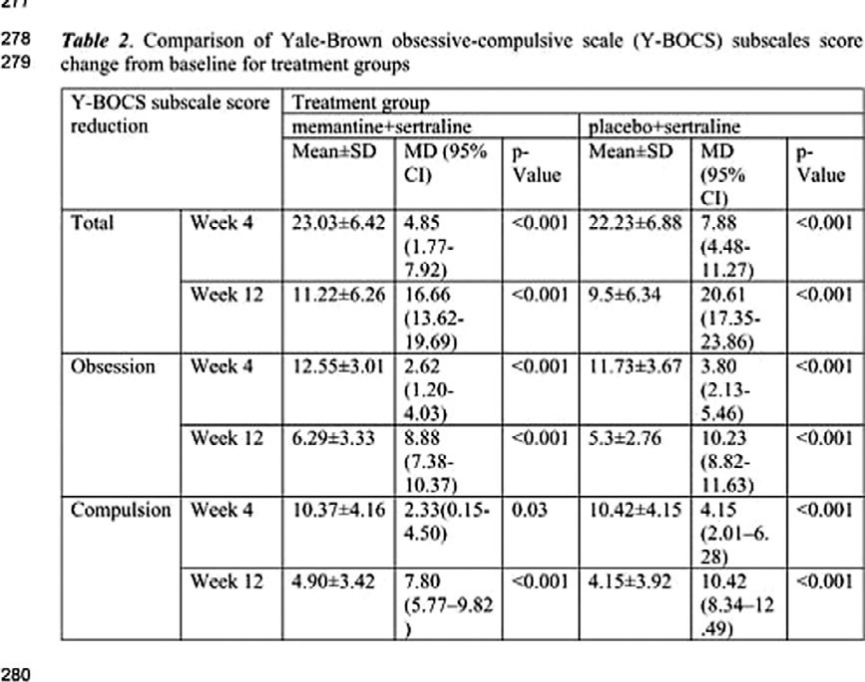

**Conclusions:**

Memantine has an acceptable safety and tolerability in patients with OCD and might have a positive effect on their executive function. Nevertheless, the current results don`t42 43 44 45 46 47 48 49 50 51 52 53 54 55 56 57 58 59 60 support the efficacy of memantine as an adjunctive agent to sertraline for symptoms in patients with OCD.

Memantine has an acceptable safety and tolerability in patients with OCD and might have a positive effect on their executive function. Nevertheless, the current results don`t support the efficacy of memantine as an adjunctive agent to sertraline for symptoms in patients with OCD.

**Disclosure of Interest:**

None Declared

